# Long shadows: a prospective study of predictors of relationship dissolution over 17 child-rearing years

**DOI:** 10.1186/s40359-014-0040-5

**Published:** 2014-10-08

**Authors:** Maren S Helland, Tilmann von Soest, Kristin Gustavson, Espen Røysamb, Kristin S Mathiesen

**Affiliations:** Department of Child Development and Mental Health, Norwegian Institute of Public Health, Postboks 4404 Nydalen, 0403 Oslo, Norway; Department of Psychology, University of Oslo, Oslo, Norway; Department of Genetics, Environment and Mental Health, Norwegian Institute of Public Health, Oslo, Norway

**Keywords:** Child-rearing, Child related strains, Longitudinal studies, Parental couple relationships, Parental dissolution and/or divorce

## Abstract

**Background:**

Parental relationship dissolutions have repeatedly been linked to negative outcomes for children, but predictors of parental dissolutions have been far less studied. Knowledge about parental dissolutions occurring after the early years of parenthood is especially sparse. The aim of the current study was to investigate whether a broad set of predictors from families of toddlers were associated with relationship dissolutions throughout the next 17 years of parenthood. We specifically investigated whether different predictors were associated with short and long term dissolutions; and whether associations with long term dissolutions were mediated by relationship dissatisfaction or child-rearing conflicts.

**Methods:**

Questionnaire data from 500 married or cohabiting mothers participating in a longitudinal population based study, the Norwegian TOPP study, was used. Child related strains, positive and negative aspects of relationship quality, and other intrinsic, environmental, and socio-demographic factors were assessed when children were 18 months old. Associations between early predictors and early dissolution (before children were 8 years old) and late dissolutions (when children were between 8 and 19 years) were compared using multinomial logistic regression analyses. Indirect paths from early predictors through relationship satisfaction and child-rearing conflicts to late dissolutions were investigated among couples that were still intact when children were 8 years old.

**Results:**

Expression of criticism and most socio-demographic variables were associated with early dissolutions only, while temperamental sociability and child related strains were associated with long term dissolutions only in the adjusted regression models. Low levels of emotional support predicted both early and late dissolutions. Associations from low emotional support and child related strains to long term dissolutions were mediated by relationship dissatisfaction, indicating that cascades towards dissolutions may originate in these early predictors. No indirect paths were identified from early predictors through child-rearing conflicts, indicating that low levels of positivity, rather than high conflict levels, are associated with dissolutions in long-term relationships.

**Conclusions:**

Predictors of dissolutions over the next 17 years could be identified among mothers of toddlers. Different predictors were associated with early and late dissolutions, indicating different cascades.

## Background

Parental dissolution rates are high across Western countries, making this perhaps the most frequent risk factor for adjustment problems during childhood. Nearly 40 percent of children in Norway experience parental dissolution during childhood (Statistics Norway 2014), and dissolution rates are even higher in the United States and other Western countries (Amato and James 2010). A large number of studies have documented an association between parental dissolution and child maladjustment (see Amato 2010 for a review). Children from dissolved families generally have more internalizing and externalizing problems, lower academic achievements and poorer social adjustment, compared with children from intact families (Frisco et al. 2007; Størksen et al. 2006; Sun and Li 2002), and the negative association between parental divorce and adjustment persist into adulthood (Amato and Sobolewski 2001; Størksen et al. 2007). Although dissolution rates vary across countries, findings have indicated that associations between family structure and child adjustment are comparable across different welfare countries, such as the United States and European and Nordic countries (Amato and James 2010; Breivik and Olweus 2006). It is important to highlight that an inference of a causal relationship between parental relationship dissolution and child maladjustment has been strongly debated, particularly because it has been difficult to investigate the association while controlling for variables that may be causes of parental dissolution as well as child maladjustment (Amato 2010). Given the extensive research attention on the negative associations between parental divorce and child adjustment, surprisingly little focus has been directed to investigating predictors of relationship dissolutions in the specific context of caring for children. A prospective study spanning both the early child-rearing years and the years with adolescent offspring may increase our understanding of how to best preserve relationships among parents, thereby also identifying risk factors needing to be controlled for in future studies of the association between parental dissolution and child maladjustment.

### Are dissolutions in the late child-rearing years associated with different predictors than short term dissolutions?

Different aspects of relationship quality may be associated with short and long term dissolutions, respectively (Gottman and Levenson 2000; Rogge et al. 2006). Broadly speaking, relationship quality can be divided into positive aspects, such as warmth, relationship satisfaction, support and positive affect; and negative aspects such as hostility, criticism, negative affect and aggression (Bradbury and Karney 2004; Rogge and Bradbury 1999). Moreover, it has been argued that the impact of positive relationship aspects may not have been given sufficient attention in former longitudinal studies of relationships (Bradbury and Karney 2004). Importantly, the impact of negative aspects may have been exaggerated due to the relatively short time span of most longitudinal studies of relationship quality and stability, as most studies have investigated newlywed couples and few have covered more than five years (Karney and Bradbury 1995). For example, Rogge and Bradbury (1999) suggested that negative aspects of relationship quality (i.e. aggression) would erode relationships more rapidly than would low levels of positivity. Consistent with this hypothesis, they found a pattern in which aggression, but not poor communication skills, predicted marital dissolution during the first five years of marriage (Rogge and Bradbury 1999; Rogge et al. 2006). However, results from a study spanning 14 years showed that low levels of positivity predicted long term dissolutions, but not short term dissolutions (Gottman and Levenson 2000). Specifically, the authors constructed and tested what they referred to as “positive and negative affect models in divorce prediction” (p. 738). They found that relationship dissolutions over the first seven years after the initial assessment were predicted by a negative affect model, including low marital satisfaction as well as expressed criticism, defensiveness, contempt, and stonewalling during a laboratory conflict conversation. Late dissolutions (i.e. between seven and 14 years after the initial assessment) were predicted by a positive affect model, including low relationship satisfaction, lack of positive affect during conflicts and thoughts of dissolution. The authors suggested that relationships characterized by intense fighting dissolve sooner than do those characterized as without positive affect, whereas in the latter type of relationships, people may become emotionally detached but postpone dissolution until the need to remain together (e.g., to raise children) becomes less crucial. Thus, predictors of short term dissolutions may not be generalizable to dissolutions in long-term relationships. This finding is yet to be replicated using different stages of parenthood as time metric.

Because parenthood is associated with different challenges at different stages, predictors of dissolutions may also differ across children’s age. The restriction of freedom in the early parenting years may be a particular important mechanism underlying the steep decline in relationship satisfaction among parents of children aged two years or lower (Twenge et al. 2003; Nelson et al. 2014). Parental couple relationships are, however, more stable than relationships between childless persons, especially during the preschool years (Rodrigues et al. 2006; Waite and Lillard 1991). It has been argued that relationship quality and commitment are the two basic motivations behind relationship dissolution (Schoebi et al. 2012). The commitment towards young children who need full attention from their parents may thus decrease the chances of leaving an unsatisfying relationship in the early parenting years. Accordingly, relatively weak associations between relationship satisfaction and divorce were found in a study following married parents over 10 years, from child age 4 to 14 (Hirschberger et al. 2009). However, low relationship quality in early childhood years may have long-term consequences for relationship dissolution when children get older and parents may feel less restraint to leave their partners. Such mechanisms have yet to be examined in long-term longitudinal studies.

It has been argued that a second high-risk period for families occur when children are entering adolescence and parents are facing their own challenges of midlife (Cherlin 2010; Steinberg 2001). Increased child autonomy may be associated with less restrictions of parental freedom, but may also be accompanied by new challenges for the couple relationship as children reach adolescence and go through pubertal development. Accordingly, relationship dissolutions are just as likely, or even more likely, to occur after the early child-rearing years, compared to parents with young children (Waite and Lillard 1991; Statistics Norway 2013; U.S. Census Bureau 2013). Few studies of parental couple relationships have covered the adolescent child-rearing years (for exceptions, see Whiteman et al. 2007; Schindler and Coley 2012; Cui and Donnellan 2009), and even fewer have spanned both the early parenting years and the years with adolescent offspring. We therefore have limited knowledge about whether predictors of dissolutions in the late child-rearing years differ from those associated with dissolutions in the early child-rearing years, and whether dissolutions in the late child-rearing years can be foreshadowed as early as when the children are toddlers.

A large body of research has highlighted the importance of investigating how aspects of relationship quality and stability are associated with intrinsic vulnerabilities and with environmental stressors couples are exposed to (Bradbury and Karney 2004; Karney and Bradbury 1995). According to the stress-vulnerability-adaption model changes in spousal relationships can be attributed to series of processes, where perceived relationship quality is influenced by each partner’s enduring strengths and vulnerabilities and by stressful events (Karney and Bradbury 1995). Empirical findings have been in line with this framework, as relationship dissolutions have repeatedly been linked to each partners’ personality characteristics such as extraversion and neuroticism (Malouff et al. 2010; Robins et al. 2002) and to depressive symptoms (Whisman and Uebelacker 2009). Knowledge is limited about to what degree such vulnerabilities and stressors may be associated with dissolution timing.

Intrinsic strengths and vulnerabilities of each partner may be involved in enduring relationship dynamics that are established early and maintained throughout the course of the relationship (Huston et al. 2001; Kelly and Conley 1987; Lavner and Bradbury 2012). Individual characteristics may thus be associated with early and late dissolutions to a different degree, due to time-varying contextual factors, such as different stages of parenthood. For instance, Kelly and Conley found that being extraverted was associated with dissolutions after 25 years of marriage, but not during the first 25 years. Extraversion is assumed to be associated with dissolutions through a mechanism where extraverted people are more socially active, thereby meeting more potential new partners (Malouff et al. 2010; Rodrigues et al. 2006). Applied to parenthood as context, it seems plausible that restrictions of freedom in the early parenting years may imply a smaller access to social arenas, involving a weaker association between extraversion and relationship dissolution in the early compared to the late child-rearing years.

Pertaining to stressors, dissolutions have been linked to living condition strains such as problems with work, housing and health (Bradbury et al. 2000; Conger et al. 1990). Associations between parental stress related to child adjustment and care-taking and relationship outcomes in the general population have however rarely been studied (Lavee et al. 1996). Studies on clinical samples have found that parenting children with a neuropsychological diagnosis was associated with divorce timing among married parents. For example, among parents of children with autism spectrum disorders divorce rates remained stable through the child-rearing years, while divorce rates decreased following the children’s early childhood (after about age 8 years) in the comparison group (Hartley et al. 2010). Pertaining to population based samples, an association between externalizing problems among three year old girls and parental divorce within the next nine years was also found in a population based study (Robbers et al. 2011), but timing of divorce was not addressed in this study. More knowledge is needed about the associations between persistent child related strains and prevalence and timing of relationship dissolutions throughout the child-rearing years.

### Are associations between early predictors and long term dissolutions mediated by relationship satisfaction or child-rearing conflicts?

It has been suggested that a cascade toward dissolution begins with declines in maternal relationship satisfaction after the arrival of the first baby (Cowan and Cowan 1995). Examining mediation effects may be useful in order to understand the mechanisms involved in long term changes in couple relationships.

Enduring strengths and vulnerabilities, early stressors, and aspects of relationship quality may be involved in a continual chain of factors, being associated with late dissolutions via long term associations with other risk factors, such as decreased relationship satisfaction or increased conflicts. Gottman and Levenson (1992) found support for a short-term cascade model, linking relationship quality to divorce over a four year period. Furthermore, they found that a composite measure involving intrinsic and environmental factors was associated with each step in their marital cascade model, indicating that a broader range of variables than relationship quality alone may be involved in cascades towards relationship dissolution. Such cascades are yet to be better understood and investigated over longer periods of time.

According to a cascade model, associations between early child-rearing stressors and relationship qualities and long term dissolutions should be mediated by intermediate relationship quality. Rogge et al. (2006) found that hostility predicted divorce during the first 4–5 years of marriage, while lack of positivity in the communication predicted low relationship satisfaction within the same time frame. The short time frame of this investigation may thus describe the beginning of a slow running cascade that may eventually lead to divorce after a longer period of time. Thus, stressors and low levels of positivity in the early child-rearing years may be associated with late child-rearing dissolutions through a cascade where low relationship satisfaction eventually causes the termination of the relationship.

Another possible cascade from early stressors and relationship quality to late child-rearing dissolutions may go through increasing child-rearing conflicts. Conflicts and negative relationship aspects may be linked to early dissolutions primarily because they are a sign of relationship deterioration, and thereby foreshadow an approaching dissolution. Thus, conflicts in the later child-rearing years may predict later dissolutions because they are more proximal to the event. An alternative cascade may thus involve an association from early child-rearing stressors and low relationship quality to conflicts or negative spousal interactions that in turn lead to relationship dissolutions in the late child-rearing years.

Adolescent child-rearing has been linked to increased negativity and conflicts between parents (Whiteman et al. 2007; Cui and Donnellan 2009). Child-rearing conflicts could be especially salient during the adolescent offspring years, because increased psychological independence from parents may increase parent–child difficulties and these may spill over to become a source of conflict between the parents (Steinberg 2001). Child-rearing conflicts among parents of adolescent have also been associated with decreased relationship satisfaction (Cui and Donnellan 2009). There are therefore compelling reasons to expect that possible associations between early child-rearing stressors and dissolutions in the late child-rearing years may be mediated by increased child-rearing conflicts.

Testing for mediation effects of both relationship satisfaction and child-rearing conflicts between the early child-rearing years and the adolescent offspring years can potentially increase our understanding of the mechanisms involved in parental dissolutions throughout the child-rearing years.

### The current study

This study examined both short and long term predictors of relationship dissolution through 17 years of parenthood among married and cohabiting mothers of toddlers. Child related strains were investigated along with a variety of other relational, intrinsic, environmental and socio-demographic predictors of relationship dissolution. Mediation effects of relationship satisfaction and child-rearing conflicts on long term dissolutions were also investigated, in order to increase our understanding of possible long term cascades towards dissolution.

Most prospective studies of couple relationships have focused on married couples, have spanned the early years of marriage and have not addressed parent specific stressors. Knowledge is therefore sparse about whether predictors of short term dissolutions can be generalized to parental dissolutions in the late child-rearing years. The present study will investigate whether a broad set of predictors among mothers of toddlers is associated with relationship dissolutions throughout the next 17 years of parenthood, using data from the population based Norwegian TOPP study. Advancing on Gottman and Levenson’s (2000) previous findings about negative and positive affect models, we will investigate whether dissolutions over the first seven year period are associated with different predictors than long term dissolutions. Dissolutions of both marriages and cohabiting unions are investigated. To capture parental couple relationships in particular, child related strains are investigated along with other predictors of relationship dissolutions. Moreover, the temporal context is organized around the age of the study child, as opposed to time of marriage. We choose to divide early and late dissolutions based on whether they occurred before or after the study child was 8 years old. This ensures that dissolutions occurring in the preschool years are grouped together, whereas all dissolutions occurring in the adolescent offspring years are analysed together in another separate group.

We will specifically investigate:

1. Whether relationship dissolutions during the late child-rearing years (from the child is 8 years and throughout adolescence) are associated with the same predictors as short term dissolutions (occurring in the preschool years and until the child is 8 years old). In accordance with the positive and negative affect models, we expect that expression of criticism will be associated with short term dissolutions, while low levels of emotional support will predict long term dissolutions. Moreover, we are aiming to identify potential timing effects of child related strains, as well as of other stressors, strengths and vulnerabilities.
2. Whether associations between early predictors and dissolutions during the late child-rearing years are mediated by relationship satisfaction or child-rearing conflicts, respectively, when the children were between 8 and 12 years, lending support to a long term cascade model.

## Methods

### Procedure

This study used data from the Norwegian longitudinal TOPP study (Tracking Opportunities and Problems). This population based questionnaire study was initiated in 1993 when mothers attending 19 different community health care centres in Eastern Norway were asked to participate with their 18 month old children. About 95% of all Norwegian families with infants and toddlers regularly attend check-ups at these health care centres. Data were collected by the staff at the health care centres again when the children were 2–3 years and 4 years old. Questionnaires were later sent by mail to mothers when the children were aged 8, 12–13, 14–15, 16–17 and 18–19 years. In the current study we include data from 18 months (T1), 8 year (T2); 12 year (T3); and 18–19 year follow-up (T4).

### Sample

Of the invited mothers, 87% (n = 913) completed the questionnaires at the first data collection. Only the 823 respondents who were initially living with the father of the index child were included in this study. Out of these, 442 responded at T2; 519 responded at T3; and 446 responded at T4, setting the response rate to 54% of the T1 sample. All respondents who participated at T4, or who had reported relationship dissolution at an earlier time point, were included in the analyses. Seven respondents were omitted from the analyses because they were widows, or had missing data regarding relationship status at T4. Altogether 500 respondents were thus included in the analyses. Out of these, 203 respondents had dissolved from their partner, whereas 297 respondents were still living with the father of the child at T4.

### Attrition and representativeness

Non-respondents at T1 did not differ significantly from respondents with respect to maternal age, education, employment status, number of children and marital status (Mathiesen et al. 1999). The dissolution rate in the sample was 41 percent over the entire period; closely resembling the dissolution rates among Norwegian parents in general (Statistics Norway 2014). At T1 the majority of respondents (70 percent) were working full or part time; six percent were students, whereas the remaining 24 percent were not working. The index child was the firstborn child in 51 percent of the families, and in 46 percent of families the index child was a boy.

Attrition from the entire TOPP-sample has been thoroughly investigated and documented (Gustavson et al. 2012). The only predictor of drop-out from 1993 to 2008 was maternal educational level. Gustavson et al. (2012) documented that the associations between variables at T1 did not differ between drop-outs and those who remained in the study, indicating that estimated associations between variables are generalizable, despite the attrition from the study.

Attrition analyses with logistic regressions based on all independent variables in the current study indicated that participants in the sample were somewhat higher educated, and had fewer children at T1, compared to non-respondents at T4 (p < .01). None of the other predictor variables were associated with drop-out.

### Measures

*Relationship dissolution* was measured at each time point by asking the mothers who they were currently living with. In addition all respondents at T4 were asked if they were still living with the father of the child and, if not, when the relationship had ended. This information was used to get more accurate information about the dissolution timing for respondents who had not participated at one or more waves between T1 and T4. A dummy variable with three values was constructed; respondents who were still living with the same partner at T4 were coded 0 (n = 297); those who dissolved between T1 and T2 were coded 1 (n = 120); and those who dissolved between T2 and T4 were coded 2 (n = 83).

*Emotional support from partner* was measured at T1 by a three item instrument pertaining to closeness, respect, and feeling of belonging, for instance “I feel closely related to my partner”. Each item had response options ranging from 1 = *completely agree* to 5 = *completely disagree* (Dalgard et al. 1995). An index was computed based on the mean of the three items. Internal consistency was *α = .*56. The average item-total correlations were above .30 which is considered satisfactory (Field 2005).

*Expression of criticism* at T1 was measured with one item where the respondents were asked to indicate how well the following statement described their relationship to their partner: “We criticize each other often”. The response options ranged from 1 = *completely agree* to 5 = *completely disagree*.

*Mothers’ psychological distress* was measured at T1 by the 25-item version of the Hopkins Symptom Check List (HSCL-25) (Derogatis et al. 1974; Hesbacher et al. 1980). Respondents were asked to indicate to what degree they had experienced a list of symptoms over the last week, such as “Feeling anxious” or “Cry easily”. Response options on all items ranged from 1 = *not at all* to 4 = *very much*. The reliability and validity of the HSCL as a measure of symptoms of anxiety and depression have been found to be good (Müller et al. 2010). Two questions (about sexual interest and suicidal thoughts) were omitted from the questionnaire in the TOPP-study because some respondents thought they were too obtrusive when they were included in a pilot. An index was computed based on the mean of the 23 items. Internal consistency was α = .90.

*Maternal temperament* was measured at T1 using the adult version of the Emotionality Activity and Sociability scale (EAS) (Buss and Plomin 1984). The factor structure and test-retest reliability of the EAS have previously been shown to be satisfactory in the TOPP sample (Nærde et al. 2004). The part of the EAS scale included in this study consists of 16 items sorted into three subscales with 12 items tapping temperamental emotionality and four items tapping sociability. Examples of items are “I often get frustrated” or “I often feel insecure” (emotionality) and “I prefer working with other people rather than alone” (sociability). Each item had response options ranging from 1 = *very typical* to 5 = *not typical*. Mean scores were computed for each subscale. Today, the terms personality and temperament are often used interchangeably (Klein et al. 2009). In essence temperamental emotionality is equivalent to the neuroticism factor of the Big Five (Clark and Watson 2008; Buss 2012), and temperamental sociability is closely associated with extraversion (Buss 2012). Internal consistencies were *α* = .54 for the sociability subscale and *α = .*74 for the emotionality subscale. The average item-total correlations were above .30 for both scales.

*Child related strains* were measured at T1 by the Child and Childcare Strain Index (Mathiesen et al. 1999). This global index has four subscales: two items pertaining to difficulties related to child care and combining work and family life; 23 items pertaining to child somatic health and illnesses; seven items tapping handicaps of the child; and 15 items tapping the child’s behaviour and adjustment to family life. The latter items were collected from the Behaviour Checklist (Richman and Graham 1971). All items were z-transformed to ensure all items to be equally weighted independent of different response options across scales when mean scores for each subscale were computed. A total score for child related strains was calculated by computing a mean score of all four subscales.

*Strains related to living conditions* were measured at T1 by a three item scale pertaining to whether the mothers experienced strains related to work (unemployment, uncertain work, difficult work relations), housing (maintenance, rental agreement etc.), and partners’ health problems (mental or somatic). The response options on each item ranged from 1 = *No* to 4 = *To a large degree* (Mathiesen et al. 1999). A composite measure was computed by calculating the mean on the three items. The two strain scales are constructed of items that measure different forms of strains, forms that will vary between life phases. Single items in formative scales are not expected to correlate. The internal consistency criteria applied to assess the quality of reflective (latent variable) scales thus do not apply to these formative (composite) scales (Bollen 1984; Mastekaasa 1987). α-values for these scales are therefore not computed.

*Socio-demographic variables.* Maternal age, number of children in the family and marital status were measured at T1. Length of mother’s education at T1 was rated from 1 = *7 years or less at school* to 8 = *4 or more years at university*. Financial resources in the family at T1 were rated from “1 - *we manage poorly*” to “5 - *we manage very well*”.

The mediator variable *relationship satisfaction* was measured with one question at T2 asking the respondents to rate their degree of satisfaction in the relationship from 1 = *very unsatisfied* to 7 = *completely satisfied*. The response rate at T2 was particularly low. In order to compensate for this, the same item at T3 was used for mothers who had not dissolved at this time point, thereby increasing the number of respondents with a valid score from n = 271 to n = 324. Specifically, a second dissolution dummy variable was computed for this purpose, indicating whether respondents had dissolved between T2 and T3. A mean score index was computed, based on satisfaction scores from both time points for those who remained together between T2 and T3, and only the T2 score for those dissolving between these time points. Internal consistency across waves was *α = .*66.

The second mediator variable, *Child-rearing conflicts,* was measured at T2 with a six item version of the Parent Problem Checklist (Dadds and Powell 1991). Each item was responded to on a five point Likert scale ranging from 0 = *almost never* to 4 = *almost always*, and mothers were asked to indicate how different issues, such as “Disagreement over household rules” or “We undermine each other” had been a problem for her and her partner over the last month. As with relationship satisfaction, the same measure at T3 was used for respondents who had not dissolved from their partner at that time point, and a mean score index for the two time points was computed. Internal consistency across waves was *α = .85.*

### Statistical procedures

All data analyses were performed with version 20 of the Statistical Package for Social Sciences (SPSS). Research question 1 was investigated by multinomial logistic regression analyses with bootstrapping. Bivariate analyses of the associations between each independent T1 variable and dissolution timing were performed first. In following multiple regression models, two sets of backward deletions were run based on the p-values of the estimates, in order to identify significant predictors of early and late dissolutions, respectively.

In investigating research question 2, indirect paths from the significant T1 predictors through T2/T3 relationship satisfaction and child-rearing conflicts to late dissolutions were tested by logistic regression analyses using the SPSS Macro Process (Hayes 2013). Sobel tests of mediation effects include a statistical assumption about normal distribution (Preacher and Hayes 2008). Because sampling distributions of indirect effects are seldom normally distributed, the Macro Process uses non-parametric bootstrapping. Bootstrapping involves repeatedly sampling from the data set and estimate the indirect effect in each resampled data set (Hayes 2013; Preacher and Hayes 2008). The 95% confidence intervals were thus estimated by drawing 5000 bootstrap samples from the original sample (each with the same n as the original sample).

### Ethics

The research is based on mothers’ questionnaire reports, given under informed consent. The research was approved by the appropriate Regional Medical Research Ethics Committee in South East Norway (REC South East).

## Results

Descriptive statistics for all independent variables and results from the one-way ANOVA performed to investigate between group differences (i.e. between those who dissolved before T2; those who dissolved between T2 and T4; and those who were still together at T4) are presented in Table 1. Between groups comparisons showed that the mean on most independent variables differed significantly between the three groups, as indicated by the significant F ratio in the one-way ANOVA analyses. Temperamental sociability and number of children were the only independent variables with no significant mean differences between the three groups.

**Table 1:** **Descriptive statistics for T1 independent variables for the total sample and couples with early dissolution, late dissolution, and no dissolution, respectively**

	**Total sample (n = 500)**			**Early dissolution (n = 120)**		**Late dissolution (n = 83)**		**No dissolution (n = 297)**		**Between groups ANOVA**
	**M**	**SD**	**Range**	**M**	**SD**	**M**	**SD**	**M**	**SD**	**F**
Emotional partner support	4.44	0.71	1-5	4.21	0.76	4.30	0.86	4.57	0.61	13.01**
Expression of criticism	2.58	1.23	1-5	2.99	1.19	2.50	1.31	2.44	1.19	8.71**
Psychological distress, HSCL	1.32	0.31	1-4	1.39	0.30	1.39	0.38	1.28	0.28	7.58**
Temperamental emotionality	2.51	0.51	1-5	2.57	0.49	2.60	0.52	2.46	0.51	3.47*
Temperamental sociability	3.80	0.58	1-5	3.80	0.61	3.89	0.55	3.78	0.57	1.11
Child related strains	0.00	0.52		0.05	0.45	0.15	0.41	−0.05	0.56	5.45**
Living condition strains	1.30	0.44		1.41	0.48	1.31	0.40	1.26	0.43	5.16**
Background variables:										
Age	30.3	4.7	19-44	28.6	5.0	29.6	4.4	31.3	4.4	16.44**
Number of children	1.65	0.65	1-6	1.61	0.79	1.52	0.65	1.70	0.82	1.58
Financial situation	3.65	0.76	1-5	3.40	0.74	3.67	0.75	3.75	0.74	9.30**
Educational length	6.27	1.37	1-8	5.84	1.34	5.96	1.32	6.52	1.34	13.41**
Marital status^a^	0.74	0.44	1-8	0.57	0.50	0.74	0.44	0.81	0.39	13.69**

^a^Marital status: 0 = *unmarried,* 1 = *married.*

*M = Mean, SD = standard deviation.*

**p < .01; *p < .05.

Inter-correlations between all independent and mediator variables are presented in Table 2. The two T1 relationship variables, emotional support from partner and expression of criticism, were moderately negatively correlated. T1 emotional support from partner was also negatively correlated with concurrent psychological distress and temperamental emotionality and with both types of strains. Expression of criticism was positively associated with psychological distress and temperamental emotionality, and with living condition strains. The strongest positive correlation was found between psychological distress and temperamental emotionality. These two predictors were negatively correlated with sociability and positively associated with child related and living condition strains. Child related strains and living condition strains were weakly positively correlated. Moreover, all predictors but temperamental sociability and three of the socio-demographic variables were significantly associated with one of, or both, mediator variables (i.e. relationship satisfaction and child-rearing conflicts). The correlations between some of the independent variables were moderate or strong, but the variance inflation factor varied between 1.07 and 1.94 for the independent and mediator variables, indicating that multicollinearity was not a problem in this sample.

**Table 2:** **Inter-correlations for independent and mediation variables (n = 500)**

**Variables**	**1**	**2**	**3**	**4**	**5**	**6**	**7**	**8**	**9**	**10**	**11**	**12**	**13**
1. T1 Emotional support	1												
2. T1 Expression of criticism	-.29**	1											
3. T1 Psychological distress, HSCL	-.38**	.25**	1										
4. T1 Temperamental emotionality	-.22**	.24**	.57**	1									
5. T1 Temperamental sociability	.08*	-.03	-.15**	-.09*	1								
6. T1 Child related strains	-.16**	.05	.20**	.13**	-.07	1							
7. T1 Living conditions strains	-.14**	.17**	.39**	.25**	-.09*	.16**	1						
8. T1 Maternal age	.04	-.03	-.13**	-.19**	-.05	-.02	-.06	1					
9. T1 Number of children	.02	-.03	.00	-.06	-.08	.06	-.02	.41**	1				
10. T1 Financial situation	.17**	-.10**	-.26**	-.14**	.08	-.13**	-.29**	.11*	-.02	1			
11. T1 Educational length	.10*	.05	-.14**	-.08	.15**	.04	.02	.25**	.06	.13**	1		
12. T1 Marital status	.12**	-.02	−.01	.03	.05	-.03	-.10*	.19**	.19**	.13**	.14**	1	
13. T2/T3 Relationship satisfaction (n = 331)	.30**	-.24**	-.20**	-.15*	.06	-.13*	-.09	-.11*	.08	.13*	.01	.06	1
14. T2/T3 Child-rearing conflicts (n = 331)	-.19**	.28**	.28**	.19**	.05	.18**	.11*	.03	-.03	-.04	.01	.02	-.33**

**p < .01; *p < .05.

Table 3 shows the results from the multinomial logistic regressions performed to investigate the associations between T1 predictors and early dissolution (prior to T2) and late dissolution (between T2 and T4), respectively. Respondents who were still together at T4 served as reference group in the analyses. Thus, an odds ratio below one indicates a negative association, whereas a ratio above one indicates a positive association between the predictor and dissolution risk. In single regression analyses both early and late dissolutions were predicted by low levels of emotional support from partner and high levels of psychological distress and temperamental emotionality. Moreover, low age and educational length predicted both early and late dissolutions. Expressed criticism, living conditions strains, being unmarried, and financial disadvantages were uniquely associated with early dissolutions. On the other hand, child related strains were uniquely associated with late dissolutions.

**Table 3:** **Multinomial logistic regression analysis for associations between T1 variables and early versus late dissolutions**

	**Early dissolutions**				**Late dissolutions**			
	**Single**		**Multiple**		**Single**		**Multiple**	
**Variables**	**OR**	**95% CI**	**OR**	**95% CI**	**OR**	**95% CI**	**OR**	**95% CI**
Emotional support from partner	0.60**	0.47 - 0.74	0.74*	0.57 - 0.94	0.66**	0.51 - 0.88	0.73*	0.55 - 0.99
Expression of criticism	1.57**	1.29 - 1.96	1.50**	1.20 - 1.98	1.05	0.78 - 1.40		
Psychological distress, HSCL	1.49**	1.17 -1.91			1.49**	1.09 - 1.98		
Temperamental emotionality	1.25*	1.00 - 1.54			1.31*	1.04 - 1.66		
Temperamental sociability	1.04	0.84 - 1.35			1.22	0.92 - 1.62	1.43*	1.07 - 1.97
Child related strains	1.25	1.00 - 1.64			1.53**	1.21 - 2.11	1.73**	1.38 - 2.35
Living condition strains	1.39**	1.14 - 1.75			1.15	0.87 - 1.49		
Age	0.52**	0.39 - 0.67	0.62**	0.44 - 0.80	0.67**	0.51 - 0.86		
Number of children	0.90	0.68 - 1.13			0.78	0.69 - 1.00		
Financial situation	0.61**	0.47 - 0.76	0.74*	0.57 - 0.96	0.89	0.68 - 1.15		
Educational length	0.58**	0.46 - 0.74	0.67**	0.50 - 0.87	0.63**	0.48 - 0.82	.59**	0.43 - 0.78
Marital status	0.58**	0.46 - 0.72	0.68**	0.57 - 0.87	0.83	0.64 - 1.10		

Early dissolutions: within T2 (child age 8 years) (n = 120); Late dissolutions: between T2 and T4 (child age 8–19 years) (n = 83).

Reference group = still together on T4 (child age 19 years) (n = 297).

95% Confidence intervals are based on bootstraps for parameter estimates. **p<.01: *p<.05.

In the adjusted models, most predictors were uniquely associated with either early or late dissolutions. Expression of criticism, low age, financial disadvantages, and being unmarried were associated with early dissolutions only. Late dissolutions were uniquely associated with temperamental sociability and child related strains. Low levels of emotional support and low educational level were associated with both short and long term dissolutions.

**Figure 1:**
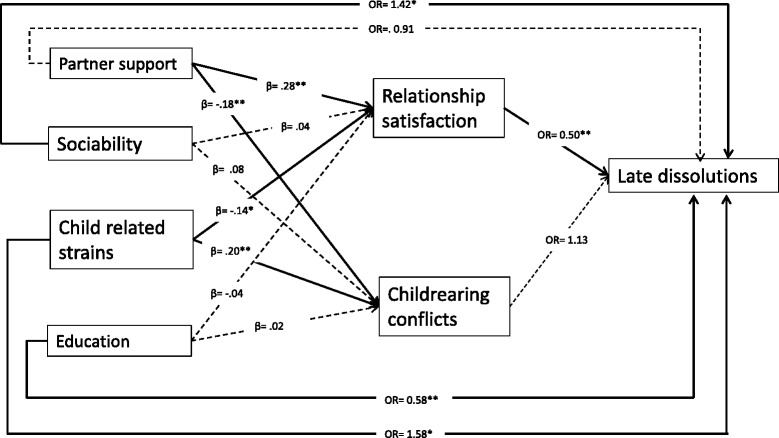
**Direct and indirect paths from T1 predictors to late dissolutions (between T2 and T4).** Note: n = 324 (59 = late dissolved; 265 = together at T4). *p < .05, **p < .01. Odds ratio for complete indirect path from partner support through relationship satisfaction: .82 (bootstrapped confidence intervals = .66 to .95). Odds ratio for complete indirect path from child related strains through relationship satisfaction: 1.10 (bootstrapped confidence intervals = 1.02 to 1.29).

Research question 2 pertained to mediations of the associations between the early predictors and late dissolutions. The results from the regression analyses of indirect paths from the significant T1 predictors through T2/T3 relationship satisfaction and child-rearing conflicts to T4 dissolutions are displayed in Figure 1. Only the predictors that were significantly associated with late dissolutions in the multiple multinomial regressions were included in these analyses. All estimates are adjusted for the other significant T1 predictors of late dissolutions. The analyses showed that relationship satisfaction, but not child-rearing conflicts mediated the relationship between partner support and late dissolutions. Partner support was significantly associated with both relationship satisfaction and child-rearing conflicts, but only relationship satisfaction was significantly associated with late dissolutions. The direct effect of emotional support from partner on late dissolution was no longer significant once T2/T3 relationships satisfaction was adjusted for. Moreover, the indirect path through relationship satisfaction was statistically significant (see Figure 1), indicating complete mediation. The indirect path from partner support through child-rearing conflicts was insignificant (OR = .98; bootstrapped CI = .90 to 1.03).

The association between child related strains and late dissolutions was partly mediated by T2/T3 relationship satisfactions, but not by child-rearing conflicts. Child related strains were significantly associated with T2/T3 relationship satisfaction which was in turn significantly associated with late dissolutions. Moreover, the complete indirect path from child related strains through relationship satisfaction to late dissolution was significant (see Figure 1). However, the direct effect of child related strains on late dissolution was still significant when T2/T3 relationships satisfaction was adjusted for, indicating that the association was only partly mediated by relationship satisfaction. Child related strains were significantly associated with child-rearing conflicts, but these did not predict late dissolutions and the indirect path from child related strains through child-rearing conflicts was insignificant (OR = 1,03; bootstrapped CI = .96 to 1.12).

The associations between T1 sociability and educational level and late dissolutions were not mediated by T2/T3 relationship satisfaction or child-rearing conflicts, as they were not significantly associated with the mediator variables.

## Discussion

The present study show that long term predictors of parental relationship dissolutions over a time span of 17 years can be detected among married and cohabiting mothers of toddlers. Early and late dissolutions were partly associated with different predictors. Expressed criticism and most socio-demographic variables predicted early dissolutions only, whereas temperamental sociability and child related strains uniquely predicted long term dissolutions in multiple logistic regressions. Low emotional support from partner predicted both short term and long term dissolutions. Associations between both child related strains and low levels of partner support and late dissolutions were mediated by relationship satisfaction, indicating long term cascades through low levels of positivity rather than through increased negativity. We emphasize the point that the unique predictors of late dissolutions would not have been detected as predictors of relationship dissolution if the time span of the current study had been considerably shorter. This highlights the importance of a long time span when investigating predictors of parental relationships dissolutions.

### Different predictors of early and late dissolutions

Different predictors were associated with dissolutions in the early and the late child-rearing years. Expression of criticism was associated with early dissolutions only. This result was in line with previous findings of negative aspects of relationship quality as especially destructive, thereby being associated with rapid deteriorations of couple relationships (Gottman and Levenson 2000; Rogge et al. 2006). We expanded on these findings by showing that these associations were also detectable in the context of parenting small children.

Low levels of emotional support predicted dissolutions both in the early and the late child-rearing years. This finding was only partly in line with Gottman and Levenson’s (2000) findings of lack of positive affect as associated with late dissolutions. They suggested an interpretation where conflict-free but passionless relationships may be sufficient for the cooperation needed to raise children, but as potentially troubling when a midlife crisis occur. Our results expanded on these findings by indicating that initial low levels of emotional support may result in relationship dissolution during the years with adolescent offspring and by showing that these associations were also evident when intrinsic strengths and vulnerabilities and stressors were adjusted for. Contrary to the findings by Gottman and Levenson, we found that low levels of emotional support were also associated with short-term dissolutions. This differing finding may be due to the different conceptualizations of negativity and positivity in the two studies. For example, Gottman and Levenson included relationship satisfaction both in their positive affect and negative affect model. Another possibility is that low levels of positivity is more detrimental when couples have to cooperate about raising children, and is therefore associated with early dissolutions as well.

The finding of temperamental sociability as a predictor of late dissolutions only was in line with previous findings of extraversion being associated with dissolutions more than 25 years into marriage, but not prior to this period (Kelly and Conley 1987). One possible interpretation of this finding may thus be that it is associated with different phases of parenthood. The restrictions of freedom are especially strong during the early child-rearing years (Twenge et al. 2003). As children grow older, mothers can spend more time on social arenas such as paid work or other out-of-home activities. Being extraverted may thus increase the chances of finding alternative partners and thereby increase the likelihood of leaving the relationship.

The finding of child related strains as associated with late dissolutions only was partly in line with the findings by Hartley et al. (2010) of married parents of children diagnosed an autism spectrum disorder, as they found that parents in the clinical sample were just as likely as parents in the control group to divorce during the early child-rearing years. The child related strains investigated in the current study may be less challenging than what is associated with having a child diagnosed with a neuropsychological disorder and this may be one reason for the different results.

Different predictors may indicate different mechanisms to be involved in dissolutions in the early and the late child-rearing years, respectively. Importantly, risk factors for dissolutions in the late child-rearing years could be detected far ahead, when the children were toddlers. Metaphorically speaking, the findings thus highlighted that “old *strains* cast long shadows”. Temperamental sociability and child related strains were uniquely associated with late dissolutions. They may therefore be referred to as ‘long shadow predictors’.

### Mediation effects on associations between early predictors and late dissolutions

Two possible long term cascades were identified, as associations between both child related strains and low levels of emotional support among parents of toddlers and relationship dissolutions in the late child-rearing years were mediated by relationship satisfaction. Gottman and Levenson (1992) presented a short term cascade model for divorces in which low relationship quality is leading to consideration of relationship dissolution, which may in turn lead to dissolution. The current findings lend support to a longer term cascade model, where initial low levels of emotional support are associated with subsequent lower levels of relationship satisfaction, eventually resulting in dissolution.

A cascade originating in initial child related strains may be related to dissolution timing. Strains related to child care-taking and children’s adaption to family life were associated with subsequent low levels of satisfaction between partners, resulting in increased dissolution risk in the late child-rearing years. One possible mechanism may be that obligations associated with having more strains related to child care-taking may act as a barrier towards leaving the relationship during the early parenting years, but a long term association with low relationship satisfaction may eventually end in dissolution when the children become increasingly independent and the commitment towards them are decreasing.

The context of parenthood may also be important for understanding a cascade towards dissolution originating in low levels of emotional support among parents of toddlers. Children are not only a potential source of strains, but may also provide a source of closeness and support for parents. Adolescent development has however been linked to changes in the parent–child relationship involving more emotional distance, less mutual acceptance and children spending less time with their parents (Steinberg 2001; Whiteman et al. 2007). Decreased closeness in the parent–child relationship may thus accompany the cascade evolving from less emotional support from partner, making the lack of support and satisfaction even more detrimental in the late child-rearing years. This study thus expanded on the previous works by Gottman and Levenson (1992) by being able to link dissolution cascades to the stages of parenthood.

Child-rearing conflicts did not mediate the associations between the early predictors and late dissolutions, as child-rearing conflicts did not predict late dissolutions. This finding is lending further support to the assumption of low levels of positivity, rather than high levels of negativity, as the main predictor of dissolutions in long-term relationships. Even though the adolescent child-rearing years have been associated with increased parental conflicts (Whiteman et al. 2007; Cui and Donnellan 2009), these may not result in relationship dissolutions once the parents have overcome the challenges of the initial child-rearing years.

The positive associations between temperamental sociability and dissolution risk were in line with previous findings of an association between extraversion and divorce (Kelly and Conley 1987; Rodrigues et al. 2006). In line with the idea of commitment and relationship quality as two different motivations for dissolutions (Schoebi et al. 2012), high levels of temperamental sociability predicted dissolutions, without being associated with relationship satisfaction or child-rearing conflicts. Temperamental sociability was however associated with dissolution risk only when other variables were adjusted for. Although high levels of sociability are associated with an increased chance of relationship dissolutions, the characteristic thus also seems to be positively associated with protective factors suppressing the effect of sociability in the unadjusted regression model. For instance, socially outgoing individuals may have more satisfied partners and have higher levels of general well-being, but may be less committed to their relationship as they have a higher access to other sources of social support.

### Strengths and limitations

While the study has strengths such as using data from a population based sample, a long follow-up period and a broad set of predictors, there are also some methodological limitations that could guide future research. Firstly, the findings rely on reports from one family member only, the mother. As a result, our conclusions only apply to maternal reports of relationship quality, vulnerabilities and stressors, not to paternal or to couple’s joint experiences. Correlations among variables may be inflated by the perspective of a single source. This limitation is due to the sampling method, as mothers were the far most frequent attendants at health care check-ups in 1993. Secondly, some measures and scales have been changed, removed or added since the TOPP-study was started in 1993 to give the advantage of better validated outcome measures. The internal reliability of some of the early predictors, particularly temperamental emotionality and emotional support from partner, was rather low. These scales consisted of few items, and this may negatively affect the α-value (Cortina 1993). Nevertheless, the somewhat low internal reliability may have caused under-estimations of the associations. Thirdly, attrition in the TOPP-study is substantial. A Monte Carlo simulation study showed that estimates of associations are far more robust to selective attrition than are estimates of prevalence and mean values, and that associations between attrition and study variables had to approach a strong effect size before estimates of associations became biased in a situation with 50 percent attrition in a an original sample of n = 1000 (Gustavson et al. 2012). Furthermore, associations between variables at T1 did not differ between respondents and drop-outs from the TOPP-study between 1993 and 2008 (Gustavson et al. 2012), suggesting that estimated associations between variables are still generalizable. Nevertheless, we do not know whether the longitudinal associations differ between those who dropped out and those who remained in the study, and the selective attrition may thus have resulted in under-estimations of associations between education, number of children and dissolution risk in the current sample.

Finally, the study was conducted on a rather homogenous population of Norwegian mothers, and future studies should try to replicate the findings in other contexts. Having a particular kind of welfare system such as in Scandinavia might affect the association between the early predictors and dissolution risk. For instance, Norway was the most gender equal country in the world according to the Human Development Index (Malik 2014). Moreover, all Scandinavian countries are characterized by liberal norms regarding single parenthood, divorce and non-marital childbearing (Kiernan 2000; Tjøtta and Vaage 2003). This being said, predictors of relationship dissolutions have generally been found to be comparable across countries (Amato and James 2010). An important direction for future research would however be to investigate whether similar timing effects of early predictors are found in other welfare contexts.

### Implications and future directions

The results showed that easily assessable predictors among mothers of toddlers were associated with both short and long term dissolutions. When children are toddlers, most mothers in Norway are returning to work, while they are still regularly in contact with the family health care centers, and stressors occurring in this period can therefore easily be detected by public services. The detection of early predictors from a questionnaire based study is particularly suitable for screening and is therefore important in regard to the potential for early detection and interventions.

Importantly, the findings indicated that mothers who experienced many child related strains or received less emotional support from their partner in the early parenting years were more likely to leave their partner as this was associated with lower satisfaction later on. Thus, parents exhibiting these signs may be potential candidates for prevention programs. For instance, in Norway a nationwide relationship education program is available for free for all couples who are having their first child (Thuen and Lærum 2005), but relatively few parents attend these programs. One important implication of the current study would thus be to better target couples who should be encouraged to attend this program. Furthermore, the findings suggest that focusing on increasing relationship satisfaction and positivity between partners may be more effective than preventing conflicts in order to prevent dissolutions.

The findings may guide future attempts to clarify the impact of parental dissolutions on child adjustment. Inferences of a causal relationship between parental relationship dissolution and child maladjustment have been strongly debated, and one important reason for this is the difficult task of investigating the association while controlling for variables that may be causes of parental dissolution as well as child maladjustment (Amato 2010). The predictors of dissolutions found in our study, may be important mediators in this association and should therefore for controlled for in future studies of parental dissolution and child adjustment.

## Conclusions

The study expanded on previous studies by showing that predictors of both short and long term relationship dissolutions through 17 years of parenthood could be detected when the children were toddlers. The time span of the current study was large, and covered almost the entire child-rearing period. During this period large changes are taking place in most families. Despite these naturally occurring changes there is a remarkable possibility of detecting signs of long term relationship dissolution risk when the children are as young as 18 months old. We will especially stress that dissolution more than seven years later was predicted by child related strains even when adjusting for earlier relationship characteristics, and that these associations were mediated by intermediate relationship satisfaction. This implies that even though parents are not exhibiting signs of concurrent relationship problems, strains related to child-caretaking may indicate that actions should be taken to prevent later relationship dissolutions.

## References

[CR1] Amato PR (2010). Research on divorce: continuing trends and new developments. Journal of Marriage and Family.

[CR2] Amato PR, James S (2010). Divorce in Europe and the United States: commonalities and differences across nations. Family Science.

[CR3] Amato PR, Sobolewski JM (2001). The effects of divorce and marital discord on adult children’s psychological well-being. American Sociological Review.

[CR4] Bollen KA (1984). Multiple indicators: internal consistency or no necessary relationship?. Quality and Quantity.

[CR5] Bradbury TN, Fincham FD, Beach SR (2000). Research on the nature and determinants of marital satisfaction: a decade in review. Journal of Marriage and Family.

[CR6] Bradbury TN, Karney BR (2004). Understanding and altering the longitudinal course of marriage. Journal of Marriage and Family.

[CR7] Breivik K, Olweus D (2006). Children of divorce in a Scandinavian welfare state: are they less affected than US children?. Scandinavian Journal of Psychology.

[CR8] Buss, AH. (2012). *Pathways to Individuality: Evolution and Development of Personality Traits *American Psychological Association.

[CR9] Buss AH, Plomin R (1984). Temperament: Early Developing Personality Traits.

[CR10] Cherlin AJ (2010). The Marriage-Go-Round: The State of Marriage and the Family in America Today.

[CR11] Clark LA, Watson D, John OP, Robins RW, Pervin LA (2008). Temperament: An organizing paradigm for trait psychology. Handbook of personality: Theory and research (3rd ed.).

[CR12] Conger RD, Elder GH, Lorenz FO, Conger KJ, Simons RL, Whitbeck LB, Huck S, Melby JN (1990). Linking economic hardship to marital quality and instability. Journal of Marriage and the Family.

[CR13] Cortina JM (1993). What is coeffiicient alpha – an examination of theory and applications. Journal of Applied Psychology.

[CR14] Cowan CP, Cowan PA (1995). Interventions to ease the transition to parenthood: why they are needed and what they can do. Family Relations.

[CR15] Cui M, Donnellan MB (2009). Trajectories of conflict over raising adolescent children and marital satisfaction. Journal of Marriage and Family.

[CR16] Dadds MR, Powell MB (1991). The relationship of interparental conflict and global marital adjustment to aggression, anxiety, and immaturity in aggressive and nonclinic children. Journal of Abnormal Child Psychology.

[CR17] Dalgard OS, Bjørk S, Tambs K (1995). Social support, negative life events and mental health. The British Journal of Psychiatry.

[CR18] Derogatis LR, Lipman RS, Rickels K, Uhlenhuth EH, Covi L (1974). The Hopkins Symptom Checklist (HSCL): a self-report symptom inventory. Behavioural Science.

[CR19] Field A (2005). Discovering Statistics using SPSS.

[CR20] Frisco ML, Muller C, Frank K (2007). Parents’ union dissolution and adolescents’ school performance: comparing methodological approaches. Journal of Marriage and Family.

[CR21] Gottman JM, Levenson RW (1992). Marital processes predictive of later dissolution: behavior, physiology, and health. Journal of Personality and Social Psychology.

[CR22] Gottman JM, Levenson RW (2000). The timing of divorce: predicting when a couple will divorce over a 14‐year period. Journal of Marriage and Family.

[CR23] Gustavson K, von Soest T, Karevold E, Røysamb E (2012). Attrition and generalizability in longitudinal studies: findings from a 15-year population-based study and a Monte Carlo simulation study. BMC Public Health.

[CR24] Hartley SL, Barker ET, Seltzer MM, Floyd F, Greenberg J, Orsmond G, Bolt D (2010). The relative risk and timing of divorce in families of children with an autism spectrum disorder. Journal of Family Psychology.

[CR25] Hayes A (2013). Introduction to Mediation, Moderation, and Conditional Process Analysis: a Regression-Based Approach. Methodology in the Social Sciences.

[CR26] Hesbacher PT, Tickels K, Morris RJ, Newman H, Rosenfeld H (1980). Psychiatric illness in family practice. Journal of Clinical Psychiatry.

[CR27] Hirschberger G, Srivastava S, Marsh P, Cowan CP, Cowan PA (2009). Attachment, marital satisfaction, and divorce during the first fifteen years of parenthood. Personal Relationships.

[CR28] Huston TL, Caughlin JP, Houts RM, Smith SE, George LJ (2001). The connubial crucible: newlywed years as predictors of marital delight, distress, and divorce. Journal of Personality and Social Psychology.

[CR29] Karney BR, Bradbury TN (1995). The longitudinal course of marital quality and stability: a review of theory, methods, and research. Psychological Bulletin.

[CR30] Kelly EL, Conley JJ (1987). Personality and compatibility: a prospective analysis of marital stability and marital satisfaction. Journal of Personality and Social Psychology.

[CR31] Kiernan K, Bachrach C, Hindin M, Thomson E, Thornton A (2000). European Perspectives on Union Formation. The Ties that Binds. Perspectives on Marriage and Cohabitation.

[CR32] Klein DN, Durbin CE, Shankman SA, Hammen CL, IH Gotlib (2009). Personality and mood disorders. Handbook of depression.

[CR33] Lavee Y, Sharlin S, Katz R (1996). The effect of parenting stress on marital quality an integrated mother-father model. Journal of Family Issues.

[CR34] Lavner JA, Bradbury TN (2012). Why do even satisfied newlyweds eventually go on to divorce?. Journal of Family Psychology.

[CR35] Malik K (2014). Human Development Report 2014: Sustaining Human Progress: Reducing Vulnerabilities and Building Resilience.

[CR36] Malouff JM, Thorsteinsson EB, Schutte NS, Bhullar N, Rooke SE (2010). The five-factor model of personality and relationship satisfaction of intimate partners: a meta-analysis. Journal of Research in Personality.

[CR37] Mastekaasa A (1987). Models, indices, and consistency criteria. Tidsskrift for Samfunnsforskning.

[CR38] Mathiesen K, Tambs K, Dalgard O (1999). The influence of social class, strain and social support on symptoms of anxiety and depression in mothers of toddlers. Social Psychiatry and Psychiatric Epidemiology.

[CR39] Müller JM, Postert C, Beyer T, Achtergarde S (2010). Comparisons of eleven short versions of the symptom checklist 90-revised (SCL-90-R) for use in the asessment of general psychopathology. Journal of Psycholpathology and Behavioral Assessment.

[CR40] Nelson, SK, Kushlev, K, & Lyubomirsky, S. (2014). The pains and pleasures of parenting: when, why, and how is parenting associated with more or less well-being? *Psychological Bulletin, ᅟ*Advance online publication. http://dx.doi.org/10.1037/a0035444. 10.1037/a0035444 24491021

[CR41] Nærde A, Røysamb E, Tambs K (2004). Temperament in adults – reliablity, stability and factor structure of the EAS temperament survey. Journal of Personality Assessment.

[CR42] Preacher KJ, Hayes AF (2008). Asymptotic and resampling strategies for assessing and comparing indirect effects in multiple mediator models. Behavior Research Methods.

[CR43] Richman N, Graham P (1971). A behavioural screening questionnaire for use with three‐year‐old Children. Preliminary findings. Journal of Child Psychology and Psychiatry.

[CR44] Robbers SC, Bartels M, van Beijsterveldt CT, Verhulst FC, Huizink AC, Boomsma DI (2011). Pre-divorce problems in 3-year-olds: a prospective study in boys and girls. Social Psychiatry and Psychiatric Epidemiology.

[CR45] Robins RW, Caspi A, Moffitt TE (2002). It’s not just who you’re with, it’s who you are: personality and relationship experiences across multiple relationships. Journal of Personality.

[CR46] Rogge RD, Bradbury TN (1999). Till violence does us part: the differing roles of communication and aggression in predicting adverse marital outcomes. Journal of Consulting and Clinical Psychology.

[CR47] Rodrigues AE, Hall JH, Fincham FD, Harvey JH, MA Fine (2006). What predicts divorce and relationship dissolution. *Handbook of divorce and relationship dissolution*.

[CR48] Rogge RD, Bradbury TN, Hahlweg K, Engl J, Thurmaier F (2006). Predicting marital distress and dissolution: refining the two-factor hypothesis. Journal of Family Psychology.

[CR49] Schindler HS, Coley RL (2012). Predicting marital separation: do parent–child relationships matter?. Journal of Family Psychology.

[CR50] Schoebi D, Karney BR, Bradbury TN (2012). Stability and change in the first 10 years of marriage: does commitment confer benefits beyond the effects of satisfaction?. Journal of Personality and Social Psychology.

[CR51] Statistics Norway (2013). Familier og husholdninger, 1. januar 2013.

[CR52] Statistics Norway. (2014). http://ssb.no/befolkning/statistikker/familie/aar/2013-04-11?fane=tabell&sort=nummer&tabell=107390S2013 [cited 2014 10.05.]

[CR53] Steinberg L (2001). We know some things: parent–adolescent relationships in retrospect and prospect. Journal of Research on Adolescence.

[CR54] Størksen I, Røysamb E, Gjessing HK, Moum T, Tambs K (2007). Marriages and psychological distress among adult offspring of divorce: a Norwegian study. Scandinavian Journal of Psychology.

[CR55] Størksen I, Røysamb E, Holmen TL, Tambs K (2006). Adolescent adjustment and well‐being: effects of parental divorce and distress. Scandinavian Journal of Psychology.

[CR56] Sun Y, Li Y (2002). Children’s well-being during parent’s marital disruption process: A pooled time-series analysis. Journal of Marriage and Family.

[CR57] Thuen F, Lærum KT (2005). A public/private partnership in offering relationship education to the Norwegian population. Family Process.

[CR58] Tjøtta S, Vaage K (2003). Union disruption in Norway. International Journal of Sociology.

[CR59] Twenge JM, Campbell WK, Foster CA (2003). Parenthood and marital satisfaction: a meta‐analytic review. Journal of Marriage and Family.

[CR60] U.S. Census Bureau (2013). Table C2: Household Relationship and Living Arrangements of Children/1 Under 18 Years, by Age and Sex: 2013.

[CR61] Waite LJ, Lillard LA (1991). Children and marital disruption. American Journal of Sociology.

[CR62] Whisman MA, Uebelacker LA (2009). Prospective associations between marital discord and depressive symptoms in middle-aged and older adults. Psychology and Aging.

[CR63] Whiteman SD, McHale SM, Crouter AC (2007). Longitudinal changes in marital relationships: the role of offspring’s pubertal development. Journal of Marriage and Family.

